# Associations between weekend catch-up sleep and health-related quality of life with focusing on gender differences

**DOI:** 10.1038/s41598-023-47244-z

**Published:** 2023-11-20

**Authors:** Jinkyung Oh, Eunmi Kim, Iksoo Huh

**Affiliations:** 1 College of Nursing, Seoul National University, Seoul, 03080 Republic of Korea; 2 The Research Institute of Nursing Science, Seoul National University, Seoul, 03080 Republic of Korea

**Keywords:** Public health, Quality of life

## Abstract

This study investigated associations between weekend catch-up sleep (WCUS) and health-related quality of life (HRQoL) in 15,837 participants from the 7th (2016–2018) Korea National Health and Nutrition Examination Survey. We categorized WCUS durations into four groups: none (≤ 0 h [h]), short (> 0 h, ≤ 1 h), medium (> 1 h, ≤ 2 h), and long (> 2 h), and performed complex samples logistic regression and likelihood ratio χ^2^ test. The study found significant associations in women for the European Quality of Life-5 Dimensions (EQ-5D) index and three EQ-5D subdomains (self-care, usual activities, and anxiety/depression) with the WCUS durations, but no significant association in men. Compared to the non-WCUS, the short or medium WCUS was positively associated with the EQ-5D index and EQ-5D subdomains (usual activities and anxiety/depression) in women, while the long WCUS significantly reduced the quality of life in the self-care domain. In an additional subgroup analysis by age, middle-aged and elderly women had a more noticeable effect of WCUS on HRQoL than young women, and the short or medium WCUS improved HRQoL in middle-aged and elderly women in general. Therefore, we recommend appropriate WCUS durations to improve HRQoL, considering both gender and age.

## Introduction

Sleep is one of the essential parts of human physiology, accounting for approximately one-third of our daily lives. Contrary to popular belief, sleep can be rather interpreted as an active process involving numerous physiological changes throughout the whole body^1^. Therefore, adequate sleep quality and quantity are fundamental to optimal physical and mental health. Besides, insufficient or excessive sleep has been associated with an increased risk of various metabolic and cardiovascular disorders, including diabetes^2^, hypertension^3^, metabolic syndrome^4^, and cardiovascular disease^5^. Additionally, sleep durations have been shown to significantly impact on cognitive and emotional domains, such as depression^6^, suicidal ideation^7^, health-related quality of life^8,9^, and cognitive functioning^10^. However, in modern society, various factors, including high-stress levels, workload, and environmental stressors, such as excessive smartphone use, can hinder individuals from obtaining the physiologically required amount of sleep.

Chronic sleep deprivation from the above various reasons results in the accumulation of sleep debt, defined as the difference between the recommended and the actual hours of sleep, and may induce detrimental health consequences^11^. Increasing the usual amount of sleep is the optimal strategy to avoid accumulating sleep debt. However, if a burden of social schedules leads to insufficient sleep during the weekdays, an alternative approach is to utilize weekend catch-up sleep (WCUS). WCUS refers to getting more sleep on weekends than on weekdays to compensate for the sleep debt accumulated during the weekdays^12^. Several studies have documented the benefits of WCUS, including improvements in obesity^13,14^, hypertension^15^, metabolic syndrome^12,16^, depressive symptoms^17^, and high sensitivity C-reactive protein^18^. Especially, researchers found that the benefits of WCUS were more significant in populations who usually sleep fewer hours^14,16,18,19^.

On the other hand, health-related quality of life (HRQoL) is a valuable metric that can provide a comprehensive understanding of the impact of WCUS on health, along with variables such as obesity and depression. HRQoL encompasses an individual’s subjective perception of their health states concerning physical, mental, and social aspects, and is a comprehensive and multifaceted measure^8^. Although previous studies have found that insufficient and excessive sleep durations can negatively influence HRQoL^8,20^, there is a lack of research examining the compensatory effects of WCUS on HRQoL.

To date, only one study has analyzed the associations between WCUS and HRQoL^19^, which reported higher HRQoL in the WCUS group than in the non-WCUS group. However, the study considered WCUS as a dichotomous variable, leaving the effect of the duration of WCUS unknown^19^. Moreover, previous studies have found that the relationship between WCUS duration and health outcomes follows a U-shaped or inverted U-shaped pattern, indicating that excessively long WCUS negatively affects health^21^. Therefore, the associations between WCUS and HRQoL also need to be examined based on the specific duration of WCUS, and we divided the WCUS durations into several groups in the association analysis accordingly, so that the specific durations for WCUS could be derived for clinical recommendations.

In addition, several studies have examined the differences in the effect of WCUS according to weekday sleep duration, chronotype, and age^12,14,16,18,19^. However, little attention has been paid to gender differences in the effect of WCUS^22,23^, despite the well-established differences in sleep patterns and sleep quality between men and women due to physiological factors^1,24–26^. Thus, our study also aims to investigate whether the associations between specific WCUS durations and HRQoL exist, and they differ by gender using data from the 7th (2016–2018) Korea National Health and Nutrition Examination Survey (KNHANES), which is a nationally representative sample.

## Results

### General characteristics of subjects by gender and WCUS durations

Table 1 shows the basic characteristics of the subjects according to the WCUS durations for men and Table 2 for women. Of the total 15,837 subjects, 6959 (43.9%) were male, and 8878 (56.1%) were female, and the proportions of the WCUS durations within each gender were as follows: non-WCUS (55.1% for men and 52.5% for women), short WCUS (18.9% and 21.4%), medium WCUS (14.9% and 15.1%), and long WCUS (11.1% and 11.0%). When examining the differences in general demographics by WCUS durations, we found significant differences in most variables regardless of gender, except BMI (*p* = 0.815) and high-risk drinking (*p* = 0.170) in the results of the men subgroup.

**Table 1:** Baseline characteristics of study participants according to the weekend catch-up sleep durations in men using adjusted one-way ANOVA for continuous variables and Rao-scott chi-square test for categorical variables.

Variables (WCUS) (h)	Non-WCUS (≤ 0 h)	Short WCUS (> 0 h, ≤ 1 h)	Medium WCUS (> 1 h, ≤ 2 h)	Long WCUS (> 2 h)	*P*-value
Total (n = 6959) (0.70 $\pm$ 0.02)	4203 (55.1)	1207 (18.9)	890 (14.9)	659 (11.1)	
Age (years)	49.84 $\pm$ 0.33	41.79 $\pm$ 0.49	39.70 $\pm$ 0.48	38.64 $\pm$ 0.55	< 0.001
Body mass index (kg/m^2^)					
< 23 (0.74 $\pm$ 0.03)	1378 (32.3)	391 (32.2)	293 (33.7)	222 (33.5)	0.815
≥ 23, < 25 (0.70 $\pm$ 0.04)	1092 (25.3)	302 (24.7)	216 (23.8)	177 (26.8)	
≥ 25 (0.67 $\pm$ 0.03)	1733 (42.4)	514 (43.1)	381 (42.5)	260 (39.7)	
Smoking status					
Never smoker (0.80 $\pm$ 0.04)	896 (23.1)	343 (30.6)	253 (31.1)	171 (26.5)	< 0.001
Past smoker (0.54 $\pm$ 0.03)	1913 (40.9)	453 (34.8)	301 (30.4)	189 (25.7)	
Current smoker (0.79 $\pm$ 0.04)	1394 (36.0)	411 (34.5)	336 (38.5)	299 (47.8)	
High-risk Drinking					
No (0.71 $\pm$ 0.02)	3376 (78.2)	978 (81.8)	694 (78.9)	520 (78.0)	0.170
Yes (0.66 $\pm$ 0.05)	827 (21.8)	229 (18.2)	196 (21.1)	139 (22.0)	
Physical activity (metabolic equivalent of task)					
< 600 (0.62 $\pm$ 0.03)	2419 (54.3)	570 (44.0)	418 (45.3)	314 (46.9)	< 0.001
≥ 600, < 3,000 (0.79 $\pm$ 0.03)	1448 (36.4)	537 (46.0)	386 (44.3)	273 (41.7)	
≥ 3,000 (0.73 $\pm$ 0.07)	336 (9.3)	100 (10.1)	86 (10.5)	72 (11.4)	
Household income					
Low (0.41 $\pm$ 0.05)	959 (18.6)	107 (7.3)	62 (8.3)	47 (7.2)	< 0.001
Low-middle (0.70 $\pm$ 0.04)	1056 (23.9)	247 (21.2)	204 (22.1)	163 (24.0)	
High-middle (0.73 $\pm$ 0.04)	1075 (28.1)	389 (33.9)	286 (32.6)	216 (32.4)	
High (0.80 $\pm$ 0.03)	1113 (29.4)	464 (37.6)	338 (37.0)	233 (36.5)	
Education level					
$\le$ Middle school (0.31 $\pm$ 0.04)	1363 (24.5)	155 (9.5)	81 (6.4)	91 (10.3)	< 0.001
High school (0.74 $\pm$ 0.04)	1393 (36.3)	396 (34.5)	320 (38.5)	250 (40.1)	
$\ge$ College (0.81 $\pm$ 0.03)	1447 (39.2)	656 (56.0)	489 (55.1)	318 (49.6)	
Marital status					
Single $(0.95\pm 0.04)$	681 (23.7)	321 (32.7)	264 (36.5)	229 (40.5)	< 0.001
Married $(0.61\pm 0.02)$	345 (6.7)	40 (2.9)	27 (2.4)	28 (3.6)	
Separated/divorced/widowed (0.42 $\pm$ 0.07)	3177 (69.6)	846 (64.4)	599 (61.1)	402 (55.9)	
Employed					
No (0.47 $\pm$ 0.04)	1453 (29.7)	220 (18.5)	120 (15.7)	89 (14.7)	< 0.001
Yes (0.77 $\pm$ 0.02)	2750 (70.3)	987 (81.5)	770 (84.3)	570 (85.3)	
Chronotype					
Morning (0.26 $\pm$ 0.03)	1057 (18.7)	108 (6.8)	46 (3.8)	40 (5.0)	< 0.001
Intermediate (0.65 $\pm$ 0.02)	2512 (61.4)	776 (63.4)	578 (62.1)	368 (52.6)	
Evening (1.03 $\pm$ 0.05)	634 (20.0)	323 (29.8)	266 (34.1)	251 (42.4)	
MSFsc (local time)	3:40 am $\pm$ 3.1 min	4:13 am $\pm$ 4.7 min	4:25 am $\pm$ 4.1 min	5:02 am $\pm$ 8.7 min	< 0.001
Weekday sleep duration (h)	7.26 $\pm$ 0.02	6.94 $\pm$ 0.03	6.61 $\pm$ 0.04	6.08 $\pm$ 0.06	0.007
WCUS duration (h)	-0.19 $\pm$ 0.01	0.81 $\pm$ 0.01	1.76 $\pm$ 0.01	3.48 $\pm$ 0.05	< 0.001

For categorical variables, values are shown as n (weighted %); for continuous variables, values are shown as weighted mean ± standard error. Except for the first row about total counts, the rest of proportions are calculated by each of columns. *h* hours, *MSFsc* mid-sleep on free days corrected for sleep debt on workdays, *WCUS* weekend catch-up sleep.

**Table 2:** Baseline characteristics of study participants according to the weekend catch-up sleep durations in women using adjusted one-way ANOVA for continuous variables and Rao-scott chi-square test for categorical variables.

Variables (WCUS) (h)	Non-WCUS (≤ 0 h)	Short WCUS (> 0 h, ≤ 1 h)	Medium WCUS (> 1 h, ≤ 2 h)	Long WCUS (> 2 h)	*P*-value
Total (n = 8878) (0.71 $\pm$ 0.02)	5050 (52.5)	1795 (21.4)	1217 (15.1)	816 (11.0)	
Age (years)	52.11 $\pm$ 0.36	44.01 $\pm$ 0.39	41.81 $\pm$ 0.44	37.67 $\pm$ 0.60	< 0.001
Body mass index (kg/m^2^)					
< 23 (0.80 $\pm$ 0.03)	2261 (47.6)	979 (55.6)	660 (56.2)	468 (58.9)	< 0.001
≥ 23, < 25 (0.71 $\pm$ 0.04)	1084 (20.2)	333 (17.4)	251 (20.4)	154 (19.1)	
≥ 25 (0.56 $\pm$ 0.03)	1705 (32.2)	483 (27.0)	306 (23.4)	194 (22.1)	
Smoking status					
Never smoker (0.71 $\pm$ 0.02)	4499 (87.7)	1615 (89.3)	1090 (89.0)	692 (84.3)	< 0.001
Past smoker (0.73 $\pm$ 0.07)	289 (6.1)	108 (6.5)	67 (5.7)	54 (6.3)	
Current smoker (0.81 $\pm$ 0.10)	262 (6.2)	72 (4.1)	60 (5.3)	70 (9.5)	
High-risk Drinking					
No (0.70 $\pm$ 0.02)	4808 (94.4)	1700 (94.5)	1145 (93.6)	741 (89.8)	< 0.001
Yes (0.90 $\pm$ 0.08)	242 (5.6)	95 (5.5)	72 (6.4)	75 (10.2)	
Physical activity (metabolic equivalent of task)					
< 600 (0.63 $\pm$ 0.02)	3215 (60.9)	988 (53.2)	632 (51.7)	415 (49.3)	< 0.001
≥ 600, < 3,000 (0.83 $\pm$ 0.03)	1648 (35.0)	725 (41.6)	521 (42.9)	357 (45.3)	
≥ 3000 (0.83 $\pm$ 0.08)	187 (4.1)	82 (5.2)	64 (5.5)	44 (5.4)	
Household income					
Low (0.40 $\pm$ 0.04)	1330 (22.9)	215 (10.8)	111 (8.4)	91 (11.5)	< 0.001
Low-middle (0.68 $\pm$ 0.04)	1318 (25.8)	397 (21.9)	277 (23.3)	191 (23.4)	
High-middle (0.74 $\pm$ 0.03)	1243 (26.7)	537 (30.6)	366 (30.7)	243 (29.2)	
High (0.89 $\pm$ 0.03)	1159 (24.6)	646 (36.8)	463 (37.6)	291 (35.9)	
Education level					
$\le$ Middle school (0.29 $\pm$ 0.02)	2304 (38.6)	365 (15.9)	208 (14.9)	112 (11.4)	< 0.001
High school (0.86 $\pm$ 0.03)	1356 (30.2)	599 (35.5)	431 (37.3)	336 (42.2)	
$\ge$ College (0.88 $\pm$ 0.03)	1390 (31.2)	831 (48.6)	578 (47.7)	368 (46.4)	
Marital status					
Single (1.30 $\pm$ 0.05)	473 (13.0)	280 (20.5)	241 (23.9)	294 (43.1)	< 0.001
Married (0.63 $\pm$ 0.02)	1223 (21.3)	213 (10.3)	121 (9.1)	67 (6.3)	
Separated/divorced/widowed (0.33 $\pm$ 0.03)	3354 (65.6)	1302 (69.2)	855 (67.0)	455 (50.6)	
Employed					
No (0.53 $\pm$ 0.02)	2784 (54.0)	713 (39.8)	457 (36.9)	252 (31.3)	< 0.001
Yes (0.87 $\pm$ 0.03)	2266 (46.0)	1082 (60.2)	760 (63.1)	564 (68.7)	
Chronotype					< 0.001
Morning (0.23 $\pm$ 0.03)	1117 (17.8)	153 (6.5)	52 (3.5)	33 (3.0)	
Intermediate (0.61 $\pm$ 0.02)	3273 (66.4)	1282 (70.0)	825 (65.8)	434 (48.7)	
Evening (1.24 $\pm$ 0.05)	660 (15.8)	360 (23.4)	340 (30.7)	349 (48.3)	
MSFsc (local time)	3:26 am $\pm$ 2.2 min	4:02 am $\pm$ 2.8 min	4:17 am $\pm$ 2.9 min	4:55 am $\pm$ 5.6 min	< 0.001
Weekday sleep duration (h)	7.29 $\pm$ 0.02	7.12 $\pm$ 0.03	6.75 $\pm$ 0.04	6.05 $\pm$ 0.05	< 0.001
WCUS duration (h)	− 0.18 $\pm$ 0.01	0.81 $\pm$ 0.01	1.75 $\pm$ 0.01	3.41 $\pm$ 0.05	< 0.001

For categorical variables, values are shown as n (weighted %); for continuous variables, values are shown as weighted mean ± standard error. Except for the first row about total counts, the rest of proportions are calculated by each of columns. *h* hours, *MSFsc* mid-sleep on free days corrected for sleep debt on workdays, *WCUS* weekend catch-up sleep.

Looking at each variable separately in terms of changing trend, as the WCUS duration increased, we found that the subjects’ average age and weekday sleep duration tended to decrease, the proportion of employed people increased, and the proportion of evening chronotype increased for both men and women. For BMI, the proportion of underweight or normal subjects tended to increase as the WCUS duration increased only in women. In terms of comparison between genders, the percentage of past and current smokers was significantly higher among men (25.7–47.8%) than women (4.1–9.5%), and the absolute percentage of high-risk drinking was also higher among men (18.2–22.0%) than women (5.5–10.2%). On the other hand, the proportion of men (9.3–11.4%) practicing a high level of physical activity was almost twice as high as women (4.1–5.5%). Among other socioeconomic characteristics, employment status showed that men (70.3–85.3%) were more likely to have a job than women, by approximately 20%p (46.0–68.7%).

### Associations between WCUS and HRQoL: Focusing on gender differences

To measure HRQoL, we used European Quality of Life-5 Dimensions (EQ-5D) and perceived health as responses. The EQ-5D contains EQ-5D index and five subdomains of the EQ-5D. Table 3 shows the gender differences in HRQoL in response to WCUS durations. No significant results were observed for HRQoL in men, while significant results were observed for the EQ-5D index and three subdomains of the EQ-5D (self-care, usual activities, and anxiety/depression) in women.

**Table 3:** Gender-wise logistic regression results for responses of health-related quality of life according to the weekend catch-up sleep durations.

Gender	Variables	Short WCUS (> 0 h, ≤ 1 h)	Medium WCUS (> 1 h, ≤ 2 h)	Long WCUS (> 2 h)	LR ${\chi }^{2}$ (*P*-value)^b^
		aOR (95% CI)^a^	aOR (95% CI)^a^	aOR (95% CI)^a^	
Men (n = 6959)	EQ-5D index	0.99 (0.74–1.32)	1.00 (0.71–1.40)	1.10 (0.75–1.63)	0.28 (0.963)
	Mobility	1.01 (0.74–1.38)	1.08 (0.72–1.60)	1.24 (0.75–2.03)	0.79 (0.853)
	Self-care	0.79 (0.44–1.41)	0.96 (0.39–2.36)	1.41 (0.57–3.49)	1.34 (0.719)
	Usual activities	1.18 (0.70–1.97)	1.03 (0.59–1.81)	1.00 (0.55–1.80)	0.42 (0.936)
	Pain/discomfort	0.84 (0.68–1.04)	1.09 (0.84–1.41)	1.09 (0.81–1.45)	4.48 (0.214)
	Anxiety/depression	0.94 (0.66–1.34)	1.28 (0.80–2.03)	0.93 (0.58–1.47)	1.85 (0.605)
	Perceived health	1.19 (0.92–1.55)	1.26 (0.96–1.66)	1.04 (0.77–1.40)	3.83 (0.280)
Women (n = 8878)	EQ-5D index	1.40 (1.14–1.71)	1.30 (1.01–1.67)	0.90 (0.67–1.19)	17.89 (< 0.001)
	Mobility	1.24 (0.98–1.56)	1.26 (0.91–1.75)	0.90 (0.62–1.29)	6.87 (0.076)
	Self-care	0.82 (0.54–1.24)	1.09 (0.60–1.97)	0.38 (0.21–0.69)	11.76 (0.008)
	Usual activities	1.44 (1.05–1.96)	1.29 (0.87–1.91)	0.79 (0.51–1.22)	9.97 (0.019)
	Pain/discomfort	1.16 (0.98–1.37)	1.11 (0.92–1.33)	0.88 (0.71–1.09)	7.56 (0.056)
	Anxiety/depression	1.28 (1.02–1.60)	1.42 (1.08–1.87)	1.18 (0.88–1.58)	9.24 (0.026)
	Perceived health	1.08 (0.90–1.30)	1.16 (0.93–1.45)	1.00 (0.78–1.29)	2.28 (0.516)

*CI* confidence interval, *EQ-5D* Euro-quality of life-5 dimension, *h* hour, *LR* ${\chi }^{2}$ likelihood ratio Chi-square statistics, *aOR* adjusted odds ratio, *WCUS* weekend catch-up sleep. ^a^Adjusted for age, household incomes, education level, marital status, employment, smoking status, physical activity, body mass index, chronotype, and weekday sleep duration; all reference group is the non-WCUS group. ^b^For LR ${\chi }^{2}$, the degree of freedom is three.

For comparisons by the WCUS durations, we estimated all adjusted odds ratios and the corresponding 95% confidence intervals based on the non-WCUS group. The adjusted odds ratio (aOR) and 95% confidence intervals for each variable are expressed as (aOR, 95% confidence interval). Specifically, in women, the short (aOR = 1.40, 1.14–1.71) or medium (aOR = 1.30, 1.01–1.67) WCUS was associated with the improved EQ-5D index. A statistical significance in the EQ-5D index was also observed for the overall effect (χ^2^ = 17.89, *p* < 0.001). In the self-care domain, a significant effect was observed only in the long WCUS, where the long WCUS significantly reduced women’s quality of life (aOR = 0.38, 0.21–0.69), and the overall effect of WCUS was also significantly associated with quality of life (χ^2^ = 11.76, *p* = 0.008). In the usual activities domain, the short WCUS significantly improved the quality of life in women (aOR = 1.44, 1.05–1.96), and the overall effect of WCUS for the domain was also significant (χ^2^ = 9.97, *p* = 0.019). Finally, in the anxiety/depression domain, the short (aOR = 1.28, 1.02–1.60) or medium (aOR = 1.42, 1.08–1.87) WCUS significantly improved women’s quality of life, and the overall effect of WCUS on the domain was also significant (χ^2^ = 9.24, *p* = 0.026).

### Associations between WCUS and HRQoL in women: Focusing on age-specific differences

In the cohort of women having associations between WCUS and HRQoL, subsequent subgroup analyses were conducted to determine any significant differences by age group (Table 4). Regarding the overall effect of WCUS in elderly women, we found a significant effect of WCUS on the EQ-5D index, perceived health, and all subdomains of the EQ-5D, except for the pain/discomfort domain. In middle-aged women, there was a significant effect of WCUS on the EQ-5D index (χ^2^ = 13.08, *p* = 0.004) and the self-care domain (χ^2^ = 16.50, *p* < 0.001), while in young women, the effect of WCUS was only significant in the anxiety/depression domain (χ^2^ = 7.84, *p* = 0.049).

**Table 4:** Logistic regression results for responses of health-related quality of life according to the age groups and the weekend catch-up sleep durations in women.

Variables	Age group^a^	Short WCUS (> 0 h, ≤ 1 h)	Medium WCUS (> 1 h, ≤ 2 h)	Long WCUS (> 2 h)	LR ${\chi }^{2}$ (*P-*value)^c^
		aOR (95% CI)^b^	aOR (95% CI)^b^	aOR (95% CI)^b^	
EQ-5D index	Young	1.17 (0.71–1.91)	0.99 (0.61–1.59)	1.13 (0.67–1.91)	0.60 (0.897)
	Middle-aged	1.52 (1.13–2.02)	1.41 (0.99–2.01)	0.87 (0.57–1.31)	13.08 (0.004)
	Elderly	1.34 (0.93–1.93)	1.76 (1.03–3.00)	0.44 (0.20–0.97)	14.40 (0.002)
Mobility	Young	0.91 (0.46–1.77)	0.69 (0.31–1.53)	0.60 (0.25–1.46)	1.50 (0.683)
	Middle-aged	1.21 (0.86–1.71)	1.24 (0.79–1.95)	0.94 (0.57–1.57)	2.53 (0.470)
	Elderly	1.34 (0.92–1.95)	1.91 (1.08–3.40)	0.67 (0.31–1.43)	9.45 (0.024)
Self-care	Young	0.30 (0.07–1.31)	0.72 (0.11–4.85)	0.87 (0.04–19.44)	2.59 (0.460)
	Middle-aged	0.65 (0.34–1.25)	2.17 (0.77–6.07)	0.25 (0.11–0.57)	16.50 (< 0.001)
	Elderly	1.67 (0.91–3.09)	0.58 (0.27–1.26)	0.29 (0.13–0.66)	12.41 (0.006)
Usual activities	Young	1.00 (0.43–2.33)	1.45 (0.59–3.55)	1.59 (0.57–4.44)	1.23 (0.746)
	Middle-aged	1.52 (0.94–2.46)	1.32 (0.69–2.52)	0.70 (0.36–1.37)	6.73 (0.081)
	Elderly	1.74 (1.14–2.65)	1.21 (0.61–2.37)	0.53 (0.24–1.16)	9.89 (0.020)
Pain/discomfort	Young	1.23 (0.88–1.72)	1.20 (0.86–1.69)	1.06 (0.72–1.54)	2.12 (0.547)
	Middle-aged	1.13 (0.91–1.39)	1.12 (0.88–1.44)	0.83 (0.60–1.15)	4.29 (0.232)
	Elderly	1.21 (0.86–1.71)	0.96 (0.59–1.57)	0.49 (0.24–0.98)	5.67 (0.129)
Anxiety/depression	Young	1.38 (0.91–2.08)	1.61 (1.01–2.55)	1.64 (1.06–2.53)	7.84 (0.049)
	Middle-aged	1.25 (0.90–1.74)	1.22 (0.83–1.80)	0.99 (0.63–1.55)	2.38 (0.498)
	Elderly	1.25 (0.81–1.93)	3.27 (1.30–8.21)	0.73 (0.32–1.69)	8.22 (0.042)
Perceived health	Young	0.92 (0.64–1.31)	1.17 (0.75–1.83)	1.10 (0.69–1.75)	1.26 (0.740)
	Middle-aged	1.13 (0.88–1.44)	1.19 (0.90–1.58)	1.03 (0.71–1.48)	2.12 (0.549)
	Elderly	1.51 (1.07–2.13)	1.08 (0.65–1.79)	0.64 (0.30–1.35)	8.10 (0.044)

*CI* confidence interval, *EQ-5D* Euro-quality of life-5 dimension, *h* hour, *LR* ${\chi }^{2}$ likelihood ratio Chi-square statistics, *aOR* adjusted odds ratio, *WCUS* weekend catch-up sleep. ^a^Age < 40 = the young (n = 2,567); ≥ 40 and < 65 = the middle-aged (n = 4218); ≥ 65 = the elderly (n = 2093). ^b^Adjusted for age, household incomes, education level, marital status, employment, smoking status, physical activity, body mass index, chronotype, and weekday sleep duration; all reference group is the non-WCUS group. ^c^For LR ${\chi }^{2}$, the degree of freedom is three.

Next, we analyzed the effect of WCUS by durations and found that the short WCUS improved the EQ-5D index (aOR = 1.52, 1.13–2.02), in middle-aged women, and the short WCUS was associated with statistically significant improvements in quality of life in the usual activities domain (aOR = 1.74, 1.14–2.65) and perceived health (aOR = 1.51, 1.07–2.13) in elderly women. In addition, the medium WCUS was associated with statistically significant improvements in the EQ-5D index (aOR = 1.76, 1.03–3.00), the quality of life in the mobility (aOR = 1.91, 1.08–3.40), and anxiety/depression domains (aOR = 3.27, 1.30–8.21) in elderly women. Finally, the long WCUS significantly reduced quality of life in the self-care domain (aOR = 0.25, 0.11–0.57) in middle-aged women, and the long WCUS was also associated with a significantly lower quality of life in the EQ-5D index (aOR = 0.44, 0.20–0.97) and self-care domain (aOR = 0.29, 0.13–0.66) in elderly women. Interestingly, for the anxiety/depression domain in young women, the medium WCUS (aOR = 1.61, 1.01–2.55) and the long WCUS (aOR = 1.64, 1.06–2.53) had a significantly positive impact on the quality of life.

## Discussion

This study divided WCUS into four durations and examined its association with HRQoL across gender and age groups. As seen in the results part, while there are no significant results in the men group, many significant results were found in the women group. In the women group, in terms of WCUS durations, the short and the medium WCUS had generally positive effects on the responses, while the long WCUS had negative effects on the responses in general. Specifically, the short WCUS significantly improved the EQ-5D index, usual activities, and anxiety/depression domains, while the medium WCUS significantly improved the EQ-5D index and anxiety/depression domains. However, the long WCUS significantly reduced the quality of life in the self-care domain. Afterwards, when subsequently examining the associations in the women group divided by age groups, we found that the middle-aged group and the elderly group showed more significant results and had more similarity in terms of directions in WCUS effects, when compared to the results of the young group. Specifically, we found that in the middle-aged women group, the short WCUS significantly improved the EQ-5D index, while the long WCUS significantly reduced the quality of life in the self-care domain. For the elderly women group, the short WCUS significantly improved the perceived health and the usual activities domain, the medium WCUS significantly improved the EQ-5D index, the mobility and anxiety/depression domains, while the long WCUS significantly reduced the EQ-5D index and the self-care domain. Interestingly, in the young women group, both the medium and the long WCUS significantly improved the quality of life in the anxiety/depression domain.

Unlike the previous study only treating WCUS as a dichotomous variable^19^, this study demonstrated the inverted U-shaped associations between the duration of WCUS and HRQoL, providing new insight into the effects of WCUS durations. These findings are also aligned with previous research and sleep hygiene guidelines related to WCUS. For example, a study by Tang et al. found a U-shaped association between WCUS and adolescent self-harm behavior^21^, supporting our findings. In addition, from a physiological perspective, not only an excessively short WCUS is not recommended due to its lack of compensatory benefits, but also an extremely long WCUS is not desirable either as it can disrupt sleep patterns and lead to delayed sleep phase syndrome, marked by notably late bedtime and wake-up time^27^. Therefore, the recommended duration of WCUS (> 0 h to 2 h) identified in this study is consistent with existing physiological sleep guidelines^28,29^. Although it is not directly related to our research topic, we additionally performed a subgroup analysis to examine the interaction between weekday sleep duration and weekend sleep duration as a supplementary analysis (Supplementary Table 1). Interestingly, the results were generally consistent, in terms of directions of effects, with existing research^14,16,19^.

Next, we found that the overall effect of WCUS was significantly stronger in women than in men. These gender differences can be attributed to women’s more significant subjective discomfort with sleep and higher rates of sleep disorders than men. Previous studies have shown that women more often report poor sleep quality and efficiency, difficulty falling asleep, daytime sleepiness, and fragmented sleep^1,24–26^. In addition, a meta-analysis highlighted that women faced a 1.41-fold increased risk of insomnia compared to men for all subjects, and the ratio rose to 1.73-fold in the women group over 65 years old^30^. In summary, our results also indirectly support existing literature highlighting poorer sleep quality in women.

In addition, researchers attribute women’s poorer sleep quality to the pronounced variability and sensitivity of their sex hormones^31^. Women experience rapid and dramatic changes in female hormone secretion throughout their life cycle, including puberty, menstruation, pregnancy, childbirth, and menopause. These hormonal changes often induce physical symptoms and changes of mood that disrupt sleep. For instance, premenstrual discomfort, menopausal vasomotor symptoms, and pregnancy-related issues like frequent urination or weight gain can impair sleep quality^32–34^. Moreover, estrogen’s role, similar to cholinergic neurotransmitters and serotonin in mood regulation, may exacerbate depressive symptoms induced by its natural decline during postpartum, premenstrual, and menopausal periods^35^, leading to a consequent reduction of sleep quality. In addition to physiological vulnerabilities, social and environmental factors such as household chores, childcare extended into the night, unfavorable socioeconomic statuses, spousal snoring, children returning home late, and traditional gender roles have also been identified as contributing to sleep disruption in women^36,37^. However, despite these physiological and socio-environmental rationales, few studies have explored the association between WCUS and gender. Considering the cross-sectional characteristics of this study, there’s a need for future longitudinal sleep studies focusing on gender-specific outcomes.

In our review of WCUS studies having gender subgroup analyses, we identified only two relevant studies. Jang et al., using data from the KNHANES, investigated the association between WCUS and hyperlipidemia in 4,085 workers from 2019–2020^22^. They found that men with 1–2 h of WCUS had a 0.64 times lower risk of hyperlipidemia than those without the sleep duration. However, this association was not significant in women. This contrast may come from the use of different outcome variables, lifestyle changes during the 2020 pandemic, and different subject criteria. Another study found associations between WCUS and subjective sleep quality with suicidal ideation and depressive symptoms in Korean adolescents^23^. It also reported more severe mental health symptoms in the subject with short WCUS and poorer sleep quality, but no significant gender difference was observed. The insignificant association between WCUS and gender may come from different sampling, data characteristics, and dependent variables. Further research is needed to find gender differences and the physiological backgrounds of the association.

Afterwards, we examined the relationship between WCUS and EQ-5D subdomains and found significant associations in women for three domains: self-care, usual activities, and anxiety/depression. These results are partially consistent with previous research^19^, which found significant effects in three domains: usual activities, anxiety/depression, and perceived health. The EQ-5D subdomains can be classified into the three categories as follows. The mobility and the self-care subdomain are into physical health, the anxiety/depression and the pain/discomfort are into mental health, and the usual activities are matched to social functioning ^19,38^. Given the characteristics of these subdomains, the results can be interpreted that the effect of WCUS is more pronounced for psychological variables, such as social functioning and mental health, so that the compensatory effect of WCUS may not sufficiently improve evaluations of physical health by itself.

Meanwhile, in the self-care domain, the long WCUS (> 2 h) was significantly associated with the lower quality of life than the non-WCUS. The result agrees with previous studies that identified an optimal WCUS duration for reducing the risk of metabolic syndrome and depression. Specifically, Son et al. reported that a duration between 1 h to 2 h was optimal for reducing metabolic syndrome risk^12^. Similarly, Kim et al. found that a duration between 1 h and 2 h was optimal for alleviating depression^17^. Among the subdomains related to physical health, self-care is an important component of the EQ-5D, in the sense that it evaluates one’s capability to carry out basic activities like grooming or personal hygiene. Although self-care is not typically linked to sleep disturbances, it is indirectly related to maintaining constant daily routines. Thus, if excessive WCUS disrupts these routines and sleep patterns, it may also significantly hinder self-care.

In the subsequent and more detailed subgroup analysis for women who showed significant associations, we found that patterns of the associations between WCUS and HRQoL are different according to the age groups. Overall, the short to medium WCUS significantly improved HRQoL including perceived health compared to the non-WCUS, while the long WCUS (> 2 h) was associated with worse HRQoL including perceived health. In addition, the benefits of WCUS on HRQoL were more pronounced among middle-aged and elderly women than young women in general. These results can be attributed to the age-associated decline in melatonin, which adversely affects average sleep duration, quality, and efficiency^39,40^. When WCUS is short or medium, it can be more beneficial to the middle-aged and the elderly groups than to the young group, because it compensates for low quality of sleep induced by low melatonin levels. However, long WCUS may significantly disrupt circadian rhythm and consequently induce reduction of sleep quality, and the groups with lower melatonin levels are more easily affected from the process^40^. Conversely, young women exhibited the improved quality of life in the anxiety/depression domain with the long WCUS. Although more precise physiological reasons are needed with further exploration, the benefits of long WCUS in young women seem to exceed the negative impacts of circadian rhythm disturbances, due to sufficient melatonin secretion. Transitions in a woman’s life, such as work, pregnancy, and childbirth, may indirectly influence these age-related differences. Hence, future sleep studies and interventions need to consider the age-specific sleep characteristics in women and suggest appropriate WCUS durations. However, as our results were based on a cross-sectional study, the causality was not conclusively proved, so further prospective studies are essential to clarify a causal relationship.

This study is meaningful as it offers initial insights into the associations between specific WCUS durations and HRQoL, highlighting gender differences. Findings in our study can serve as a foundation for future research to identify subjects who are more likely to benefit from WCUS for improved HRQoL (middle-aged and elderly women) and to recommend an appropriate duration of WCUS for them. In addition, the dataset used in this study is a large, representative sample from the KNHANES, which enhances the generalizability of the results.

Despite these strengths, our study has several limitations that need to be acknowledged. Firstly, there are debates in objectiveness of the self-reported sleep durations. Several studies reported they can have measurement bias^41,42^, while other studies have confirmed the validity of subjective sleep duration^43,44^. For more precise measurements, the use of actigraphy or smartwatches would be a solution, although it can be infeasible in the large-scale survey-based sleep studies^16,17,19,22,23^. Secondly, the large sample size of the KNHANES dataset facilitates generalization to the whole population, but the cross-sectional design of the dataset does not guarantee causal inferences. While there are some studies that identified causal effects of WCUS on neuro-mental functioning and mortality^45,46^, the research on quality of life remains sparse. Future studies need to employ prospective designs to detect more precise causal relationship between WCUS and HRQoL. Thirdly, the results of the subgroup analysis may need careful generalization and interpretation. The subgroup analysis can be advantageous when effects of covariates differ by gender and age group^47^, and this approach has been employed in many of previous sleep studies^12,19,22,23,48,49^. However, the approach usually focused on differences in significance between groups without providing statistical tests for comparison of effects between groups. While our subgroup analysis approach showed similar results, in terms of directions of effects, to those in the additional interaction analysis results (Supplementary Table 2), significant outcomes were reduced to a smaller number of domains, such as the EQ-5D index, self-care domain, and perceived health. Considering that the interaction analysis may need four times more sample size than analysis of main effects to achieve same power^50^, our results from the subgroup analysis need to be interpreted conservatively. Finally, the KNHANES dataset lacked a variety of sleep-related variables, such as sleep disorders or subjective sleep quality. Subsequent studies should consider a range of sleep-related variables, both in terms of sleep duration and qualitative factors such as sleep quality, to understand the association better.

In conclusion, our study found significant associations between WCUS and HRQoL in women. Specifically, the short or medium WCUS duration (> 0 h to ≤ 2 h) positively impacts on women's EQ-5D index and EQ-5D subdomains (usual activities and anxiety/depression) compared to the non-WCUS. However, the WCUS exceeding 2 h was associated with the reduced quality of life in women's self-care domain compared to the non-WCUS. Notably, the overall effect of WCUS was more pronounced in middle-aged and elderly women than in young women. These findings suggest that an appropriate WCUS duration needs to be recommended based on the gender and age of the subjects to improve their HRQOL.

## Methods

### Study participants and data

In this study, we selected survey participants from the 7th (2016–2018) KNHANES. The KNHANES is a cross-sectional sample survey conducted annually by the Korea Disease Control and Prevention Agency. It is a nationally representative and reliable survey using stratified cluster sampling at the household level. Clusters, the primary sampling unit, are 192 survey districts across the country selected each year considering geographical distribution. Next, for the secondary unit of sampling, 23 sample households are selected within each survey district using systematic sampling method, taking into account stratification variables (housing type, residential area ratio, etc.)^51,52^. For each sampled household, trained surveyors conduct health surveys, physical examinations, and nutrition surveys to all household members aged 1 year and older who meet the eligibility requirements. Among the surveys, the health and nutrition surveys are conducted using self-administered questionnaires^52^. Subsequently, weights are assigned to each individual subject, considering the clusters and stratification variables to ensure that the sampled data are representative of the characteristics of the whole population in South Korea. As our study is a secondary data study utilizing public data, the institutional review board of S University (IRB No. E2212/004–005) exempted our study from review. In addition, the raw data used in this study are publicly available and de-identified to ensure subject anonymity.

**Figure 1:**
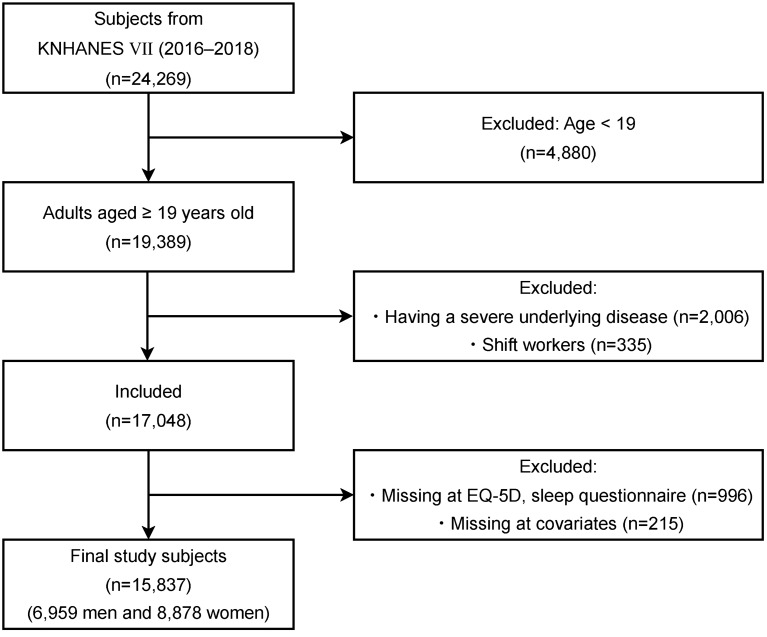
Flow chart of the filtering process of the study participants. *EQ-5D* Euro-quality of life-5 dimension, *KNHANES* Korean national health and nutrition examination survey. Diseases that strongly affect sleep patterns or health-related quality of life were excluded, such as stroke, myocardial infarction, angina, osteoarthritis, rheumatoid arthritis, all types of cancer, kidney failure, liver cirrhosis, and depression.

From a total of 24,269 subjects in the 7th wave (2016–2018), we excluded 4880 subjects under 19 years old, 2006 subjects with underlying medical conditions that could significantly impact on sleep patterns or HRQoL, and 335 shift workers^53^. Exclusion criteria included stroke, myocardial infarction, angina pectoris, osteoarthritis, rheumatoid arthritis, cancer, renal failure, cirrhosis, and depression, based on the previous study^19^. Then we excluded the 1211 subjects with missing data on the main study variables (variables of HRQoL and sleep durations) or covariates^54–56^, and finally obtained 15,837 subjects (6959 men and 8878 women) in the study (Fig. 1).

### Sleep-related variables

To calculate the values for the sleep-related variables, we used responses to the questions "On a weekday (or working day), what time do you go to bed and what time do you get up?" and "On a weekend (or non-working day, the day before a non-working day), what time do you go to bed and what time do you get up?". Then we defined WCUS as sleeping more hours on weekends than during the weekday, calculated by weekend sleep duration minus weekday sleep duration. Therefore, the value of WCUS can be negative if the weekday sleep duration is greater than the weekend sleep duration. We then divided the duration of WCUS into four categories. The first was the ‘non-WCUS’ group, whose weekday sleep duration was equal to or greater than their weekend duration (0 h or less). For subjects with WCUS, we categorized the durations into three groups: short WCUS (> 0 h to 1 h), medium WCUS (> 1 h to 2 h), and long WCUS (> 2 h)^12^. Weekday sleep duration was calculated as the difference between the time of going to bed and waking up during the weekday.

Next, we calculated the subject’s sleep–wake pattern to obtain a chronotype according to the formula proposed by the Munich Chronotype Questionnaire^57^. Specifically, we first calculated Sleep Duration on Workdays (SDW), Sleep Duration on Free days (SDF), and Mid-Sleep on Free days (MSF). Among the measures, mid-sleep refers to the time of deepest sleep and is the median of the time of going to bed and waking up. Then, using the values from the three measures, we calculated the corrected weekend sleep (Mid-Sleep on Free days Corrected for sleep debt on workdays [MSFsc]), a metric that reflects chronotype, using the formula below^57^.

$${MSF}_{sc}=MSF-0.5\times \left[SDF-\frac{(SDW\times 5+SDF\times 2)}{7}\right]$$

The MSFsc values were then divided into five groups using quintiles and finally categorized into three chronotypes. In detail, we set the morning chronotype to the first group (having the lowest MSFsc values), the intermediate chronotype to the second, third, and fourth groups, and the evening chronotype to the fifth group (having the highest MSFsc values)^58^.

### Health-related quality of life

For the HRQoL measures, we used the EQ-5D and the perceived health as responses. The EQ-5D contains the EQ-5D index and the five subdomains of the EQ-5D. Among them, the five subdomains of the EQ-5D (mobility, self-care, usual activities, pain/discomfort, and anxiety/depression) were assessed with three categories of responses. In this study, respondents who answered ‘extreme problems’ or ‘some problems’ were regarded as ‘have a problem’, and those who answered ‘no problem’ were regarded as ‘no problem’, referring to the study by Oh et al.^19^. Then each of the EQ-5D subdomains was reverse-coded as 0 for having a problem and 1 for not having a problem, implying that high scores indicate high quality of life.

The EQ-5D index is a single quantified value that is evaluated from a linear combination with the previous five subdomains. In this study, we applied the Korean version of the quality-weighted correction score for its evaluation^59^. As a result, the EQ-5D index is equal to 1 if all five subdomains are answered as ‘no problem’. Based on previous studies, the EQ-5D index was divided into quintiles and analyzed in two groups: below the first quintile (very poor) and above the first quintile^60^. We also defined perceived health as one of five responses to the question, "How do you usually feel about your health?". In this study, very good, good, and fair were categorized as ‘not poor’, and poor and very poor were categorized as ‘poor’^19^.

### Covariates

Demographic variables (age, household income, education, marital status, and employment) and lifestyle variables (smoking, alcohol consumption, and physical activity) were collected through a self-administered questionnaire. Age was subdivided into three categories: young, middle-aged, and elderly, with cutoff points of 40 and 65 years, following previous studies focused on HRQoL^61^. BMI was calculated by dividing the measured weight (kg) by the square of the height (m^2^). Then we classified it into three categories: underweight or normal (< 23), overweight (≥ 23 to < 25), and obese (≥ 25), based on the Korean Society of Obesity guidelines^62^ and criteria from the previous study^63^. Household income was categorized into four categories based on quartiles: low, low-middle, high-middle, and high, and the level of education was classified into middle school or lower, high school, and college or higher. Marital status was categorized into single, married, and the other one including separated/divorced/widowed, and employment status was categorized into employed and not employed.

Smoking status was categorized into never smoker, past smoker, and current smoker. For alcohol consumption, high-risk drinkers were defined as those who drank more than twice a week with an average alcohol consumption of 7 or more drinks for men and 5 or more drinks for women in the past year according to the criteria of the KNHANES^64^. Physical activity was assessed using the Global Physical Activity Questionnaire (GPAQ), and each subject’s physical activity level was converted to a Metabolic Equivalent of Task (MET). MET quantifies the energy per unit of body weight expended in one minute at rest. In this study, MET, which is a continuous value, was categorized into three levels according to the World Health Organization’s guidelines: low (< 600), moderate (≥ 600 to < 3000), and high (≥ 3000)^65^.

### Statistical analyses

To use the 7th KNHANES, we combined data from three years (2016–2018) by considering the complex sample weight for each year. As the statistical program, we used R 4.2.1 and all analyses including descriptive statistics were conducted using the functions (svydesign, svyglm, and svychisq) in the ‘survey’ package and for complex sample design data analysis to account for clustering, stratification variables, and weights^66,67^. The significance level for all statistical tests was set at 0.05 under a two-tailed test, and the specific analysis methods were as follows. First, to compare participants’ general characteristics and HRQoL according to WCUS durations in each gender group, we used one-way ANOVA adjusted for complex samples designed dataset to continuous variables and the Rao-Scott χ^2^ test to categorical variables^68^. Descriptive statistics were presented as weighted means and standard errors for continuous variables, and unweighted frequencies and weighted percentages for categorical variables. Second, during the complex samples logistic regression analysis, essential sociodemographic variables (age and gender) were fixed in the model at first. Then stepwise variable selection approach was conducted to identify significant covariates for each dependent variable. Afterwards, we combine all covariates selected in at least one dependent variable analysis result and use them as final covariates. With the final covariates, we conducted complex samples logistic regression analyses again to estimate the effects of WCUS duration and likelihood ratio χ^2^ tests to estimate the overall effect of WCUS^66,67^.

### Supplementary Information

Supplementary Tables.

## Data Availability

The data used in this study were obtained from the 7th (2016–2018) KNHANES from the Korea Disease Control and Prevention Agency, Ministry for Health and Welfare, Republic of Korea. All data can be freely downloaded from the official website (https://knhanes.kdca.go.kr/).
