# Supplementary material for: Associations between weekend catch-up sleep and health-related quality of life with focusing on gender differences
Source: Sci Rep. 2023 Nov 20;13:20280. doi: 10.1038/s41598-023-47244-z (PMC10662263; doi:10.1038/s41598-023-47244-z)
Supplement: Supplementary file 1 — Supplementary Tables. [file 41598_2023_47244_MOESM1_ESM.pdf]

Supplementary Tables

Title: Associations between weekend catch-up sleep and health-related quality of life with focusing on gender differences

Authors: Jinkyung Oh[1], Eunmi Kim[1], and Iksoo Huh[1,2*]

[1] College of Nursing, Seoul National University, Seoul 03080, Republic of Korea

[2] The Research Institute of Nursing Science, Seoul National University, Seoul 03080, Republic of Korea

[*] **Corresponding author:**

Iksoo Huh, Ph.D.

Associate professor, College of Nursing and the Research Institute of Nursing Science

Seoul National University

103 Daehak-ro, Jongno-gu, Seoul 03080, Republic of Korea

Email: huhixoo1@snu.ac.kr

Phone: +82-2-740-8840

Weekday sleep duration	Variables	Short WCUS (>0 h,≤1 h)	Medium WCUS (>1 h,≤2 h)	Long WCUS (>2 h)	LR χ^2
		aOR (95% CI)[a]	aOR (95% CI)[a]	aOR (95% CI)[a]	(*P*-value)[b]
≤ 6 h (n= 4,289)	**EQ-5D index**	1.46* (1.04–2.06)	1.21* (0.86–1.71)	1.17* (0.82–1.67)	5.25 (0.154)
	Mobility	1.45* (0.99–2.12)	1.32* (0.86–2.04)	1.14* (0.77–1.71)	4.35 (0.226)
	Self-care	0.70 (0.36–1.35)	0.54 (0.24–1.23)	0.98* (0.41–2.35)	3.16 (0.367)
	Usual activities	1.36* (0.83–2.24)	1.10 (0.64–1.89)	1.07* (0.62–1.87)	1.54 (0.674)
	Pain/discomfort	1.14* (0.88–1.47)	1.19* (0.91–1.56)	1.19* (0.92–1.53)	2.69 (0.442)
	Anxiety/depression	1.26* (0.84–1.90)	1.65* (1.11–2.46)	1.21* (0.82–1.78)	6.34 (0.096)
	**Perceived health**	0.93 (0.71–1.23)	1.51* (1.11–2.06)	1.32* (0.98–1.79)	10.31 (0.016)
> 6 h (n= 11,548)	**EQ-5D index**	1.12 (0.91–1.38)	1.10 (0.86–1.41)	0.93 (0.67–1.29)	2.19 (0.534)
	Mobility	1.05 (0.85–1.31)	1.07 (0.79–1.45)	1.01 (0.65–1.57)	0.40 (0.941)
	Self-care	0.79 (0.52–1.20)	1.66 (0.89–3.11)	0.36 (0.20–0.66)	15.49 (0.001)
	Usual activities	1.25 (0.89–1.77)	1.14 (0.75–1.74)	0.88 (0.53–1.47)	2.43 (0.488)
	Pain/discomfort	0.96 (0.82–1.12)	1.04 (0.86–1.25)	0.84 (0.65–1.07)	2.56 (0.465)
	Anxiety/depression	1.07 (0.87–1.33)	1.20 (0.90–1.61)	1.18 (0.81–1.72)	2.11 (0.549)
	**Perceived health**	1.17 (0.97–1.41)	1.04 (0.85–1.29)	0.90 (0.69–1.16)	3.96 (0.266)

Supplementary Table 1-A. Logistic regression results for reponses of health-related quality of life according to the weekday sleep durations and the weekend catch-up sleep durations in the total sample. *CI* confidence interval, *EQ-5D* Euro-quality of life-5 dimension, *h* hour, *LR* χ^2 likelihood ratio Chi-square statistics, *aOR* adjusted odds ratio, *WCUS* weekend catch-up sleep. [a]Adjusted for age, sex, household incomes, education level, marital status, employment, smoking status, physical activity, body mass index, chronotype, and weekday sleep duration; all reference group is the non-WCUS group. [b]For LR χ^2, the degree of freedom is three. The asterisk (*) in some cells represent higher odds ratios in the short weekday sleep duration group (≤ 6 h) than in the long weekday sleep duration group (> 6 h).

**Variables**	**Weekday sleep duration[a]**	**Short WCUS (>0 h, ≤1 h) aOR (95% CI)[b]**	**Medium WCUS (>1 h, ≤2 h) aOR (95% CI)[b]**	**Long WCUS (>2 h) aOR (95% CI)[b]**	**LR χ^2 (*P*-value)[c]**
**EQ-5D index**	≤6 h	1.31 (0.85–2.00)	1.25 (0.83–1.88)	1.01* (0.65–1.56)	2.47 (0.481)
	>6 h	1.38 (1.09–1.74)	1.25 (0.90–1.74)	0.81 (0.55–1.20)	12.07 (0.007)
Mobility	≤6 h	1.31* (0.81–2.10)	1.75* (1.01–3.02)	0.97* (0.60–1.55)	5.36 (0.147)
	>6 h	1.18 (0.90–1.54)	1.05 (0.69–1.59)	0.77 (0.45–1.32)	3.26 (0.354)
Self-care	≤6 h	0.71 (0.32–1.55)	0.91 (0.35–2.37)	0.74* (0.26–2.07)	1.10 (0.776)
	>6 h	0.77 (0.47–1.29)	1.28 (0.60–2.73)	0.22 (0.10–0.48)	16.24 (0.001)
Usual activities	≤6 h	1.49* (0.79–2.84)	1.36* (0.74–2.50)	1.03* (0.54–1.96)	2.40 (0.494)
	>6 h	1.32 (0.93–1.89)	1.18 (0.69–2.00)	0.67 (0.35–1.30)	5.12 (0.163)
Pain /discomfort	≤6 h	1.33* (0.95–1.86)	1.18* (0.84–1.67)	1.04* (0.74–1.48)	3.24 (0.356)
	>6 h	1.08 (0.89–1.31)	1.06 (0.84–1.33)	0.78 (0.57–1.06)	4.83 (0.185)
Anxiety /depression	≤6 h	1.09 (0.68–1.74)	1.39* (0.88–2.19)	0.98 (0.62–1.54)	2.25 (0.523)
	>6 h	1.31 (1.02–1.70)	1.37 (0.97–1.94)	1.65 (1.03–2.66)	9.47 (0.024)
**Perceived health**	≤6 h	0.94 (0.66–1.32)	1.57* (1.08–2.28)	1.31* (0.89–1.92)	7.31 (0.063)
	>6 h	1.11 (0.90–1.37)	0.97 (0.74–1.27)	0.85 (0.60–1.20)	2.36 (0.501)

Supplementary Table 1-B. Logistic regression results for responses of health-related quality of life according to the weekday sleep durations and the weekend catch-up sleep durations in women. *CI* confidence interval, *EQ-5D* Euro-quality of life-5 dimension, *h* hour, *LR* χ^2 likelihood ratio Chi-square statistics, *aOR* adjusted odds ratio, *WCUS* weekend catch-up sleep. [a]weekday sleep duration ≤ 6 h (n=2,435); weekday sleep duration > 6 h (n=6,443). [b]Adjusted for age, household incomes, education level, marital status, employment, smoking status, physical activity, body mass index, chronotype, and weekday sleep duration; all reference group is the non-WCUS group. [c]For LR χ^2, the degree of freedom is three. The asterisk (*) in some cells represent higher odds ratios in the short weekday sleep duration group (≤ 6 h) than in the long weekday sleep duration group (> 6 h).

Variables	Weekday sleep duration[a]	Short WCUS (>0 h,≤1 h) aOR (95% CI)[b]	Medium WCUS (>1 h,≤2 h) aOR (95% CI)[b]	Long WCUS (>2 h) aOR (95% CI)[b]	LR χ^2 (*P*-value)[c]
**EQ-5D index**	≤6 h	1.63* (0.90–2.94)	1.17* (0.62–2.19)	1.31* (0.73–2.38)	2.74 (0.434)
	>6 h	0.80 (0.58–1.11)	0.91 (0.61–1.37)	1.12 (0.63–1.99)	2.18 (0.536)
Mobility	≤6 h	1.65* (0.81–3.34)	0.97 (0.48–1.97)	1.23 (0.61–2.50)	2.19 (0.534)
	>6 h	0.84 (0.59–1.19)	1.12 (0.69–1.82)	1.56 (0.68–3.54)	2.60 (0.457)
Self-care	≤6 h	0.63 (0.16–2.41)	0.22 (0.05–0.85)	1.99* (0.39–10.27)	7.68 (0.053)
	>6 h	0.74 (0.38–1.44)	2.84 (0.76–10.57)	0.88 (0.32–2.45)	3.78 (0.286)
Usual activities	≤6 h	1.04 (0.42–2.59)	0.88 (0.34–2.28)	0.91 (0.33–2.52)	0.13 (0.988)
	>6 h	1.11 (0.59–2.10)	1.08 (0.53–2.20)	1.24 (0.57–2.71)	0.38 (0.944)
Pain /discomfort	≤6 h	0.87* (0.57–1.33)	1.22* (0.75–1.97)	1.38* (0.90–2.11)	3.93 (0.269)
	>6 h	0.79 (0.61–1.01)	0.99 (0.72–1.36)	0.89 (0.60–1.33)	3.61 (0.307)
Anxiety /depression	≤6 h	1.54* (0.76–3.11)	1.96* (0.89–4.34)	1.42* (0.69–2.91)	3.28 (0.351)
	>6 h	0.73 (0.49–1.09)	0.97 (0.57–1.68)	0.73 (0.39–1.36)	3.07 (0.382)
**Perceived health**	≤6 h	0.92 (0.60–1.41)	1.45* (0.88–2.38)	1.26* (0.79–2.03)	3.61 (0.307)
	>6 h	1.26 (0.92–1.73)	1.15 (0.82–1.61)	0.94 (0.63–1.40)	2.82 (0.420)

Supplementary Table 1-C. Logistic regression results for responses of health-related quality of life according to the weekday sleep durations and the weekend catch-up sleep durations in men. *CI* confidence interval, *EQ-5D* Euro-quality of life-5 dimension, *h* hour, *LR* χ^2 likelihood ratio Chi-square statistics, *aOR* adjusted odds ratio, *WCUS* weekend catch-up sleep. [a]weekday sleep duration ≤ 6 h (n=1,854); weekday sleep duration > 6 h (n=5,105). [b]Adjusted for age, household incomes, education level, marital status, employment, smoking status, physical activity, body mass index, chronotype, and weekday sleep duration; all reference group is the non-WCUS group. [c]For LR χ^2, the degree of freedom is three. The asterisk (*) in some cells represent higher odds ratios in the short weekday sleep duration group (≤ 6 h) than in the long weekday sleep duration group (> 6 h).

Dimension	Non-WCUS (≤ 0 h)	Short WCUS (> 0 h, ≤ 1 h)	Medium WCUS (> 1 h, ≤ 2 h)	Long WCUS (> 2 h)	LR Chisq (*p*)	df
	Beta (p-value)					
**EQ-5D index**						
Middle-aged (ref. Young)	-0.59 (<.001)				11.66 (<.001)	1
Elderly (ref. Young)	-1.34 (<.001)				62.94 (<.001)	1
Age group (overall)					66.86 (<.001)	2
WCUS	0	0.13* (.598)	-0.08* (.742)	0.02* (.931)	0.55* (.909)	3
Middle-aged*WCUS	0	0.37* (.206)	0.55* (.068)	-0.01* (.966)	4.50* (.213)	3
Elderly*WCUS	0	0.17* (.578)	0.64* (.066)	-0.81* (.089)	9.63** (.022)	3
Age group*WCUS (overall)					10.93* (.091)	6
**Mobility**						
Middle-aged (ref. Young)	-0.91 (<.001)				13.15 (<.001)	1
Elderly (ref. Young)	-1.86 (<.001)				71.73 (<.001)	1
Age group (overall)					80.07 (<.001)	2
WCUS	0	-0.11* (.751)	-0.31* (.419)	-0.29* (.457)	0.92* (.821)	3
Middle-aged*WCUS	0	0.42* (.261)	0.67* (.121)	0.43† (.335)	3.15* (.368)	3
Elderly*WCUS	0	0.38* (.325)	0.91* (.051)	-0.16* (.778)	5.32* (.150)	3
Age group*WCUS (overall)					6.78* (.342)	6
**Self-care**						
Middle-aged (ref. Young)	-1.52 (.008)				5.03 (.025)	1
Elderly (ref. Young)	-2.05 (<.001)				16.42 (<.001)	1
Age group (overall)					14.91 (<.001)	2
WCUS	0	-1.23* (.096)	-0.29* (.743)	-0.05* (.967)	3.15* (.369)	3
Middle-aged*WCUS	0	1.02† (.209)	1.37* (.190)	-0.86* (.491)	3.74* (.291)	3
Elderly*WCUS	0	1.71** (.039)	-0.25* (.797)	-1.22* (.335)	8.14** (.043)	3
Age group*WCUS (overall)					13.48** (.036)	6
**Usual activities**						
Middle-aged (ref. Young)	-0.55 (.060)				2.54 (.111)	1
Elderly (ref. Young)	-1.22 (<.001)				25.00 (<.001)	1
Age group (overall)					25.74 (<.001)	2
WCUS	0	-0.09† (.839)	0.24* (.597)	0.13* (.792)	0.45* (.929)	3
Middle-aged*WCUS	0	0.60* (.234)	0.15* (.792)	-0.30* (.597)	2.14* (.543)	3
Elderly*WCUS	0	0.64* (.191)	-0.08† (.888)	-0.73* (.215)	4.32* (.229)	3
Age group*WCUS (overall)					4.24* (.645)	6

Dimension	Non-WCUS (≤ 0 h)	Short WCUS (> 0 h, ≤ 1 h)	Medium WCUS (> 1 h, ≤ 2 h)	Long WCUS (> 2 h)	LR Chisq (*p*)	df
	Beta (p-value)					
**Pain/discomfort**						
Middle-aged (ref. Young)	-0.24 (.044)				3.56 (.059)	1
Elderly (ref. Young)	-0.57 (<.001)				26.45 (<.001)	1
Age group (overall)					17.26 (<.001)	2
WCUS	0	0.15* (.357)	0.08* (.622)	-0.05† (.752)	1.52* (.678)	3
Middle-aged*WCUS	0	0.02* (.903)	0.13* (.534)	0.01† (.974)	0.42* (.936)	3
Elderly*WCUS	0	0.05* (.842)	-0.18* (.526)	-0.66* (.083)	4.39* (.222)	3
Age group*WCUS (overall)					4.29* (.637)	6
**Anxiety/depression**						
Middle-aged (ref. Young)	-0.01 (.950)				0.04 (.846)	1
Elderly (ref. Young)	0.05 (.803)				6.02 (.014)	1
Age group (overall)					0.18 (.914)	2
WCUS	0	0.27* (.202)	0.36* (.120)	0.28* (.186)	3.97* (.265)	3
Middle-aged*WCUS	0	0.01* (.983)	-0.07† (.810)	-0.17* (.574)	0.46* (.929)	3
Elderly*WCUS	0	-0.09† (.767)	0.76* (.145)	-0.55* (.246)	4.41* (.220)	3
Age group*WCUS (overall)					4.27* (.640)	6
**Perceived health**						
Middle-aged (ref. Young)	-0.47 (<.001)				8.81 (.003)	1
Elderly (ref. Young)	-0.67 (<.001)				22.76 (<.001)	1
Age group (overall)					17.80 (<.001)	2
WCUS	0	-0.15* (.396)	0.07* (.755)	-0.04† (.854)	1.15* (.765)	3
Middle-aged*WCUS	0	0.31* (.138)	0.17* (.491)	0.14* (.600)	2.04* (.564)	3
Elderly*WCUS	0	0.57** (.019)	-0.01† (.972)	-0.37* (.387)	8.07** (.045)	3
Age group*WCUS (overall)					7.83* (.251)	6

Supplementary Table 2. Logistic regression results for responses of health-related quality of life with the interaction effects between the age groups and the weekend catch-up sleep durations in women. *EQ-5D* Euro-quality of life-5 dimension, *h* hour, *LR* χ^2 likelihood ratio Chi-square statistics, *WCUS* weekend catch-up sleep. Please note that the estimates of effects in this table are log-transformed odds ratios, while the table 4 contains raw odds ratios. In comparison to the odds ratio results in Table 4, cells with an asterisk (*) represent beta values in the same direction but not significant, cells with double asterisks (**) represent significant values in the same direction, and cells with a dagger (†) represent values in the opposite direction.
